# Unraveling the DNA Methylation in the rDNA Foci in Mutagen-Induced *Brachypodium distachyon* Micronuclei

**DOI:** 10.3390/ijms23126797

**Published:** 2022-06-18

**Authors:** Adrianna W. Bara-Halama, Dominika Idziak-Helmcke, Jolanta Kwasniewska

**Affiliations:** Plant Cytogenetics and Molecular Biology Group, Faculty of Natural Sciences, University of Silesia in Katowice, Jagiellonska 28, 40-032 Katowice, Poland; adriannabara@gmail.com (A.W.B.-H.); dominika.helmcke@us.edu.pl (D.I.-H.)

**Keywords:** *Brachypodium distachyon*, DNA methylation, FISH, maleic acid hydrazide, micronuclei, rDNA

## Abstract

Many years have passed since micronuclei were first observed then accepted as an indicator of the effect of mutagens. However, the possible mechanisms of their formation and elimination from the cell are still not fully understood. Various stresses, including mutagens, can alter gene expression through changes in DNA methylation in plants. In this study we demonstrate for the first time DNA methylation in the foci of 5S and 35S rDNA sequences in individual *Brachypodium distachyon* micronuclei that are induced by mutagenic treatment with maleic acid hydrazide (MH). The impact of MH on global epigenetic modifications in nuclei and micronuclei has been studied in plants before; however, no in situ analyses of DNA methylation in specific DNA sequence sites are known. To address this problem, we used sequential immunodetection of 5-methylcytosine and fluorescence in situ hybridization (FISH) with 5S and 25S rDNA probes on the non-dividing cells of *B. distachyon*. Such investigations into the presence or absence of DNA methylation within specific DNA sequences are extremely important in plant mutagenesis in the light of altering gene expression.

## 1. Introduction

In eukaryotic cells, chromatin exists in two forms: as loosely structured euchromatin and condensed heterochromatin. The access of mutagens to chromatin depends on the state of chromatin condensation. Chromatin structure is an essential element in the control of gene expression. The organization of chromatin, i.e., the degree of its condensation, strongly depends on the post-translational modification of histones and DNA methylation [1]. Previous studies have shown that epigenetic modifications, such as DNA methylation and histone modifications, change gene expression without modifying the DNA sequence and participate in the organism’s response to various environmental factors, including those having a mutagenic effect [2,3,4]. In the case of abiotic stresses, such as salinity, drought, heavy metals, heat, and cold, DNA hypo- or hypermethylation depends on the type of stress, its intensity or dose, and the plant species subjected to the stress [2,5].

One of the stress factors for plant organisms is mutagens. The genotoxic effects of mutagens in all eukaryotes could be observed at the gene/DNA, chromosome, and organism level [6]. Cytogenetic tests are commonly used to detect the clastogenic effects of mutagens in plant chromosomes. Among these tests, the micronucleus test is widely used for the detection and quantitative analysis of the effects of mutagens. Micronuclei (MN) are small outside-nuclear bodies located next to the interphase nucleus. They can arise from acentric chromosome fragments due to single- and double-strand breaks in DNA or from entire chromosomes due to the action of a mutagenic agent on the microtubules [7].

Maleic acid hydrazide (MH) is a commonly used chemical mutagen in plant mutagenesis [7]. MH is a uracil isomer that integrates into DNA and inhibits DNA synthesis, although the exact mechanism of its action is still not well understood. Its potential to cause chromosomal aberrations, including the formation of MN, is well documented in plant cells. Its genotoxic activity was previously confirmed in many plant species including *Hordeum vulgare*, *Allium cepa*, *Vicia faba*, and *Brachypodium distachyon*, in which the formation of MN has been observed [8,9,10,11]. MH has also been used in studies of epigenetic modifications in the response of plant cells to stress factors [3,12]. MH and gamma radiation change the level of various global epigenetic modifications, including DNA methylation, in barley cells. Interestingly, gamma radiation strongly influenced changes in the level of DNA methylation, while changes in histone methylation and acetylation were mainly noticed after treatment with MH [3]. Studies on DNA methylation and other epigenetic modifications after mutagenic treatments were carried out on the single *B. distachyon* nuclei and micronuclei [12]. The level of DNA methylation in the nuclei after mutagenic treatment differed from the level of DNA methylation present in control nuclei and strongly depended on the type of applied mutagen, MH or N-nitroso-N-methylurea (MNU). Differences in the 5-methylcytosine (5mC) levels in MH- and MNU-induced micronuclei were also observed. Our previous research suggested that DNA methylation could potentially have an active role in MN formation or be a marker for chromatin exclusion into MN [12].

Previous studies have demonstrated that the distribution of chromosome aberrations is not random. Micronuclei preferentially comprise particular chromosomes or their parts [13,14,15,16], which may be related to chromosome size, gene density [17], and other aspects of chromatin organization [18]. The hot spots of MH-induced DNA damage within the nuclei could be detected using multicolor fluorescence in situ hybridization (FISH). Among repetitive DNA sequences, ribosomal DNA (rDNA) sequences are widely used as probes for FISH in plant cells, mainly due to their availability and evolutionarily conserved nature. In *B. distachyon*, the application of rDNA sequences as probes for FISH showed that 5S-rDNA-bearing chromosomes are involved in MN formation more frequently than 35S-rDNA-bearing chromosomes [19]. The application of rDNA sequences as probes for FISH has also been useful in determining the origin of micronuclei in other plant species, e.g., barley [6,10,20]. It has been shown that the micronuclei of some plant species [21] and humans [22] are more often formed with the participation of nucleolar organizing regions. These cytogenetic approaches improve the sensitivity of the MN test.

Until now, the DNA methylation of particular DNA sequences or chromosome regions, both in the cell nucleus and micronucleus, has not been analyzed in single plant cells within the context of mutagenesis. Such an approach would provide further useful information about the possible mechanisms that govern MN formation and elimination followed by mutagenic treatment in plants. This strategy has not yet been successfully applied to studies of micronuclei in plants.

Here, for the first time, we demonstrate the DNA methylation in the foci of 5S and 35S rDNA sequences in the individual *B. distachyon* micronuclei that are induced by mutagenic treatment with MH. Although the impact of MH on the level of global epigenetic modifications has been studied in plants, to date no in situ analyses regarding the rDNA sites are known. For this purpose, immunodetection of 5-methylcytosine and FISH with 5S and 25S rDNA probes were applied sequentially to the nuclei and micronuclei of *B. distachyon* cells treated with MH.

## 2. Results

### 2.1. The Presence of rDNA Signals in MH-Induced B. distachyon Micronuclei

**Figure 1:**
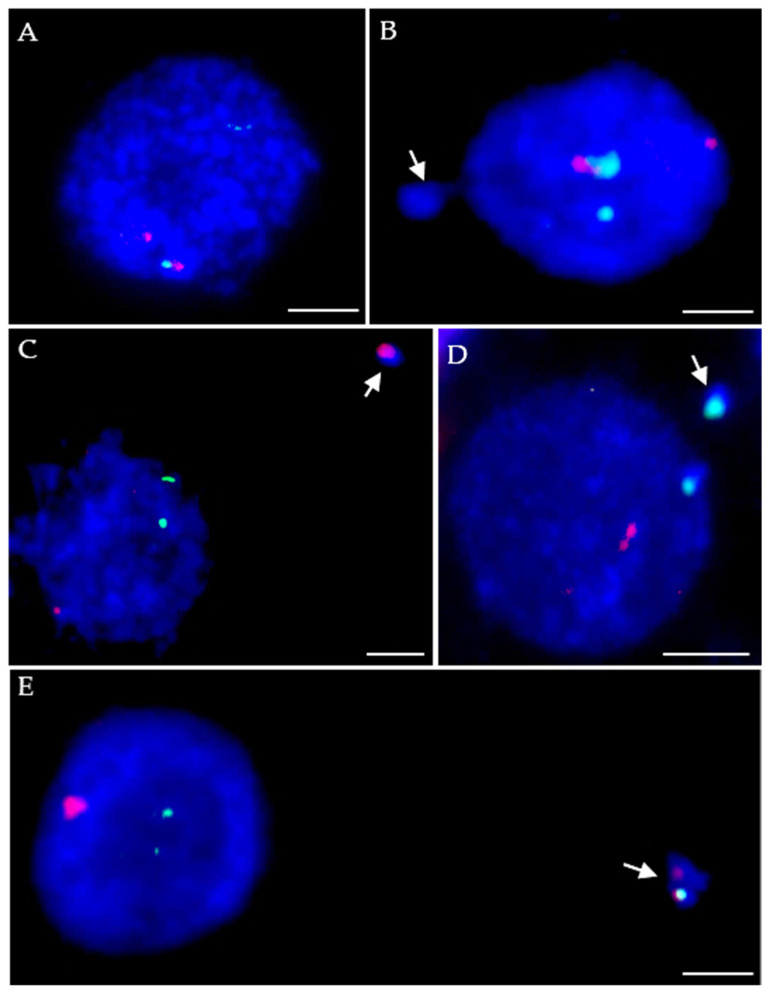
*B. distachyon* interphase nuclei: control (**A**) and with micronuclei (MN) induced by MH treatment (**B**–**E**), FISHed with 5S rDNA (red) and 25S rDNA probes (green). The chromatin was stained with DAPI (blue). Arrows indicate the micronuclei. Types of micronuclei are described in the main text. Scale bars = 5 µm.

Fluorescence in situ hybridization (FISH) with 5S and 25S rDNA as probes was applied to the interphase nuclei of *B. distachyon* plants treated with 4 mM MH and control (not-treated) plants. The examples of control nuclei and nuclei with micronuclei from MH-treated cells are shown in Figure 1.

**Figure 2:**
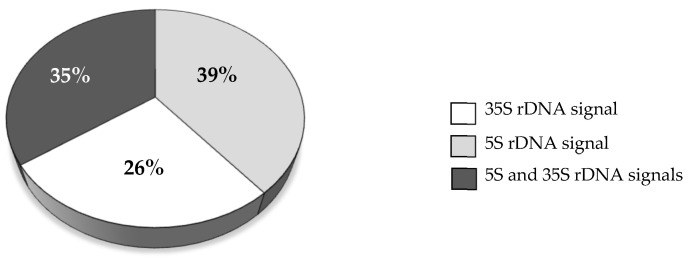
The frequencies of MN induced by MH treatment displaying signals of 5S and/or 35S rDNA. The results obtained after different post-treatment times, 0 h, 10 h, 20 h, are combined into one dataset.

In the control nuclei two signals from 5S rDNA and two signals from 35S rDNA were observed (Figure 1A), which corresponds to the number of rDNA loci that are present in *B. distachyon* genome. In the case of MH-treated nuclei with MN, four situations were discerned: (1) no rDNA signal in MN; (2) one 5S rDNA signal in MN; (3) one 35S rDNA signal in MN; and (4) both 5S and 35S rDNA signals in MN (Figure 1B–E, respectively). We analyzed the frequencies of MN with signals of 5S and/or 35S rDNA in *B. distachyon* cells subjected to different post-treatment times: 0 h, 10 h, 20 h, following MH treatment (Figure 2).

No differences in the frequencies of MN with rDNA signals were observed with regard to the post-treatment time, so the results have been combined into one dataset. MN with 5S rDNA signals were observed with the highest frequency of 39%, whereas MN with both 5S and 35S rDNA foci had a slightly lower frequency of 35%. MN with 25S rDNA signals only were the least frequent (26%).

### 2.2. The Presence of 5S and/or 35S rDNA and 5mC Signals in B. distachyon Micronuclei

**Figure 3:**
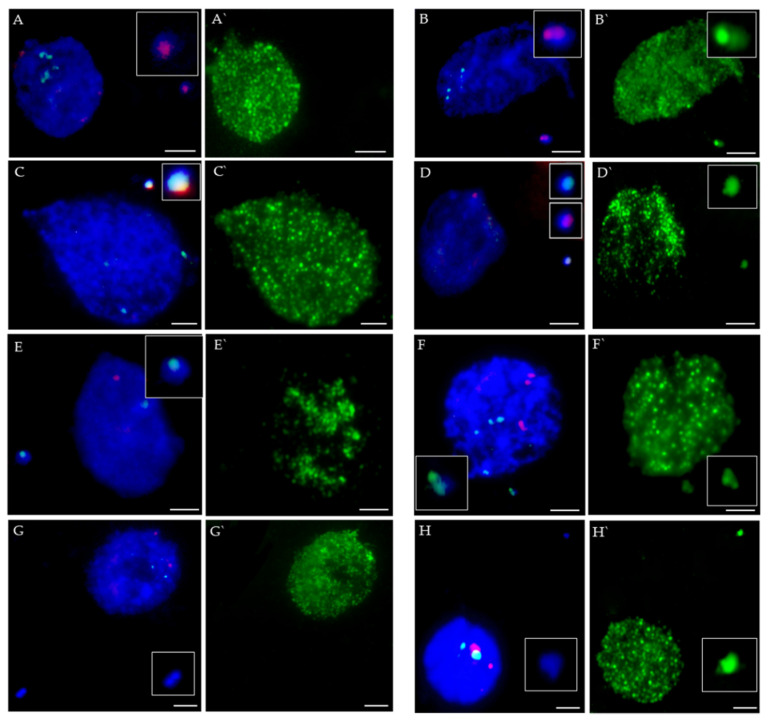
*B. distachyon* interphase nuclei with MN, FISHed with 5S rDNA (red) and 25S rDNA probes (green), stained with DAPI (blue) (**A**–**H**), and subjected to the immunocytochemical detection of 5mC (green) (**A`**–**H`**). Types of micronuclei are described in the main text. Small images of magnified micronuclei are shown in each picture. Scale bars = 5 µm.

The FISH with 5S and 25S rDNA as probes and immunocytochemical detection of 5-methylcytosine (5mC) applied to the same nuclei have enabled the analysis of the occurrence of DNA methylation precisely in the rDNA sites (Figure 3). Based on the diversity of signals we distinguished two main categories of MN with rDNA: with 5mC signals (Figure 3B,B`,D,D`,F,F`) and without 5mC signals (Figure 3A,A`,C,C`,E,E`). The MN without rDNA foci are shown in Figure 3G,H. The last group could also be divided into two subsets: with and without 5mC signals (Figure 3G`,H`, respectively).

**Figure 4:**
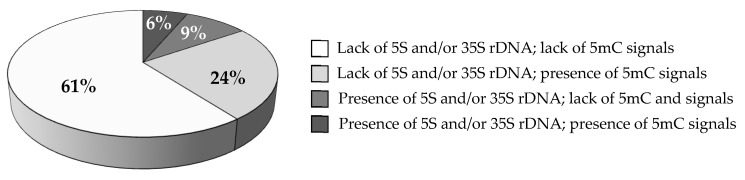
The frequencies of MN induced by MH treatment classified into different groups related to the presence or lack of both rDNA and 5mC signals. The results obtained after different post-treatment times, 0 h, 10 h, 20 h, are combined into one dataset.

The frequencies of MN with different signal compositions were analyzed. Figure 4 presents the frequencies of MN with different signals after different post-treatment times combined into one dataset. Among all analyzed MN, only 15% had the signals of rDNA. Within this group, 41% of MN had 5mC signals within these foci. The MN lacking both the presence of rDNA and 5mC foci constituted 61% of the total MN number, while the frequency of MN displaying 5mC foci but no rDNA signals was 24% (Figure 4).

**Figure 5:**
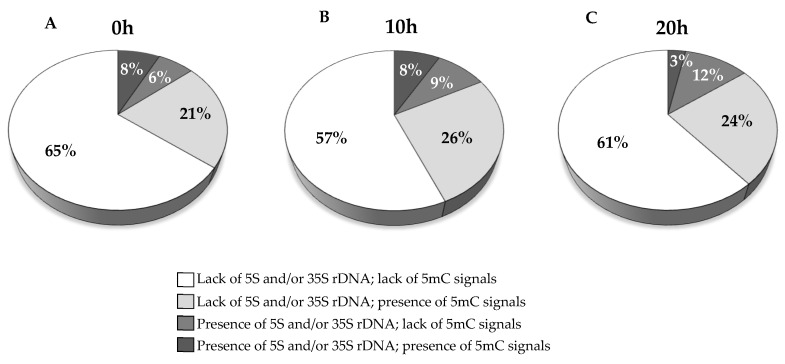
The frequencies of MN induced by MH treatment classified into different groups related to the presence or lack of rDNA and 5mC signals. The results for (**A**) 0 h, (**B**) 10 h, (**C**) 20 h of post-treatment time are presented.

We observed mostly minor differences in the frequency of MN with specific signal composition between nuclei subjected to different post-treatment lengths (Figure 5). The MN lacking the rDNA signals dominated in all cases, with rates of 86%, 83%, and 85% for 0 h, 10 h, and 20 h of post-treatment, respectively. Interestingly, we noted a significant difference in the frequencies of MN with and without 5mC signals in the rDNA regions (3% and 12%, respectively) only in the MN subjected to 20 h of post-treatment. In contrast, only slight differences in the rates of rDNA-bearing MN with and without DNA methylation foci were observed after 0 h and 10 h of post-treatment. 

### 2.3. Differences in DNA Methylation of 5S and 35S rDNA Foci in B. distachyon Micronuclei

**Figure 6:**
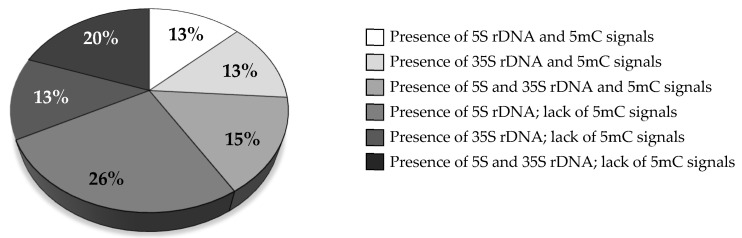
The frequencies of MN induced by MH treatment, with rDNA foci classified into different groups related to the presence or lack of 5mC signals. The graph shows the results for the different types of rDNA separately. The results obtained after different post-treatment times, 0 h, 10 h, 20 h, are combined into one dataset.

The MN displaying rDNA signals were subjected to a more detailed analysis in order to investigate the differences in DNA methylation between 5S and 35S rDNA foci in MN. We compared the frequencies of MN bearing either one or both rDNA signals, which were additionally immunostained with anti-5mC antibodies conjugated with Alexa 488 (Figure 6).

We observed that the frequency of MNs carrying only 5S rDNA without 5mC signals in this region was two times higher than of those with 5mC signals (26% vs. 13%, respectively). In contrast, no differences in terms of the presence or absence of 5mC signals were observed in the MN bearing only 35S rDNA foci. In both cases the frequencies equaled 13%. The rates of the presence or absence of 5mC signals in MN with both 5S and 25S rDNA signals were relatively similar and equaled 15% and 20%, respectively.

## 3. Discussion

Micronuclei (MN) are considered to be hallmarks of genome instability. Despite the long tradition of using the MN test [23], the mechanism of the formation and elimination of micronuclei is still unknown. The occurrence of MN is commonly attributed to a failure in the repair of DNA double-strand breaks (DSB). A number of studies on human MN indicate that the contribution of particular chromosomes or their parts to MN is not entirely random [24,25], suggesting the existence of ‘hot spots’ more prone to DSB. The spatial organization of the nucleus at interphase, the diverse transcriptional activity of specific chromosome regions, chromosome size, or the presence of large heterochromatin blocks are among the factors that could be responsible for the non-random distribution of DSB that lead to MN formation [26].

The identification of the origin of MN in plants is possible by using FISH with repetitive DNA sequences, such as centromeric, telomeric, and rDNA, as probes. In our experiment only 15% of all MN displayed rDNA signals. This result confirms that 5S rDNA and 35S rDNA loci are not “hot spots” for DNA breaks in the *B. distachyon* genome after the application of MH, as was previously stated by Kus et al. [27].

The 5S rDNA site is localized proximally in the long arm of chromosome Bd4, while a nucleolar organizing region with transcriptionally active 35S rDNA loci is found distally in the short arm of chromosome Bd5 [28]. The interstitial localization of the 5S rDNA site means that two breaks in the double-stranded DNA flanking the site would be required in order to incorporate the 5S rDNA sequence into MN. In comparison, in the case of the terminally located 35S rDNA site, only one DSB is necessary. Considering only chromosomal position, one should expect that 35S rDNA will be more sensitive to mutagens than 5S rDNA. Remarkably, our study demonstrated that MN with 5S rDNA signals were observed at a higher frequency than MN with 35S rDNA. Kus et al. [19] also showed that 5S-rDNA-bearing chromosomes were involved in *B. distachyon* MN formation more frequently than 35S-rDNA-bearing chromosomes. Similar differences in the sensitivities of 5S and 35S rDNA to MH treatment were previously reported in *Hordeum vulgare* [29] and *Crepis capillaris* [30,31]. In the latter species, the application of comet–FISH demonstrated that 5S rDNA was present in the comet tail more frequently than 35S rDNA. These phenomena can be explained by the association of the active 35S rDNA with the nucleolus [30,32]. The authors postulated that 35S rDNA, which represents housekeeping genes, associated with the nucleolus, is less prone to fragmentation and migration into the comet tail. The combination of the MN test with analysis of the transcriptional activity of 35S rRNA genes in MH-treated *H. vulgare* showed that the expression of 35S rDNA is always maintained in MN, even though they are eliminated during the next cell cycle [29].

A number of studies have shown that epigenetic DNA modifications play a key role in gene expression under different stresses in plants. Although epigenetic regulation is not associated with changes in the nucleotide sequence, it can lead to heritable changes in gene expression [33]. DNA methylation can protect the plant genome from a variety of mutations [34], for example by deactivation of transposable elements [35]. Very recently epigenetic modifications in response to abiotic stresses such as high or low temperature, high salt exposure, and deficient or flood water conditions over generations has been reported [36,37,38,39]. Natural variation in DNA methylation associated with environmental changes was observed for example in tomato and soybean [40,41]. Epigenetics has attained great success for its applications in plant breeding, where it has been used to assess the propagation of epigenetic marks across generations to improve desirable crop traits [42].

However, the correlation between epigenetic modifications and changes that are induced by mutagens is still not well understood. Most recent research on DNA methylation relies on molecular and biochemical techniques. For the determination of methylation in the DNA molecule, cloning and sequencing are widely used methods that may even provide single-nucleotide resolution of methylation. Newly developed high-throughput sequencing tools for identifying RNA alterations have greatly advanced the practical study of RNA epitranscriptomics [43,44]. The methodical approach applied in this paper, based on the detection of methylated sites in situ on cell nucleus preparations, is not common, but in some studies, e.g., those aimed at explaining the mechanism of micronucleus formation, it is the only method.

However, in some respects, complementary cytological studies are necessary to analyze the 5mC distribution in situ in plant chromosomes and nuclei [45,46]. Examining the distribution of highly methylated chromosomal regions by using antibodies directed against 5mC enables an insight into the correlation between the instability of the genome and its structure at the cytological level. Such analyses appear to be particularly important in studies on the effects of mutagens that manifest themselves as micronuclei.

There is evidence suggesting that increased chromatin condensation may play a role in DNA elimination from the nucleus. In the crosses between wheat and pearl millet, uniparental genome elimination of pearl millet occurs during the early stages of embryo development. The pearl millet chromosomes form micronuclei, which are generally more condensed than the nuclei, and contain either heterochromatin and euchromatin or exclusively heterochromatin [47]. In sciarid flies, the paternal chromosome set undergoing elimination shows low levels of histone H4/H3 acetylation, which is usually an indicator of heterochromatinization [48]. Another hallmark of heterochromatin, histone H3K9 methylation, is required for the chromatin elimination that accompanies the development of the somatic macronucleus in *Tetrahymena* [49]. In our case, the presence of 5mC signals was noted in only 30% of the micronuclei, suggesting that heterochromatinization might not play such an important part in the DNA exclusion resulting from the mutagenic treatment. However, cytosine methylation is only one of heterochromatin’s signature features, and other markers, such as the level of histone H3K9 and H3K27 methylation, should be involved in future studies on micronuclei formation.

We show that among all MN that were bearing rDNA, 41% had 5mC signals within these foci. Our results can be interpreted in relation to the DNA methylation patterns in the *B. distachyon* submetacentric chromosomes Bd4 and Bd5, which carry 5S and 35S rDNA loci, respectively. Individual *B. distachyon* chromosomes exhibit a characteristic DNA methylation pattern, with numerous differences in the distribution of the methylated sites between homologous chromosomes as well as between the arms of a given chromosome [28]. However, some chromosome sites, such as pericentromeric regions, are invariably highly methylated in all chromosomes. Chromosomes Bd4 and Bd5 have characteristic 5mC foci distribution. In the case of chromosome Bd4, two peaks with a high density of 5mC foci are observed with a slight decrease in the proximal region of the long arm carrying 5S rDNA locus. A survey of methylation patterns in Arabidopsis indicates that highly repetitive sequences, such as rDNA sequence arrays, are usually densely methylated [50,51]. The high DNA methylation level is often correlated with transcriptional inhibition. 5S rDNA is an exception to that rule since it remains an active genetic region despite being highly methylated [51]. We found that unmethylated 5S rDNA sequences participated in MN formation two times more often than methylated 5S rDNA. Since a decrease in DNA methylation is linked to its decondensation, such a result might indicate that the unmethylated, less-condensed 5S rDNA sites are more likely to suffer from mutagen-induced DSB. This hypothesis can be corroborated by the results of studies on human lymphocytes, which demonstrated that the decondensation of heterochromatic regions on chromosomes 1 and 9 caused by idoxyuridine treatment strongly correlated with MN formation by these chromosomes [52].

The 5S rDNA sequence has been reported to have a higher methylation and condensation level than 35S rDNA in Arabidopsis [53] and tobacco [54]. 5S rDNA also appeared to be more resistant to hypomethylation in Brassica [55]. However, despite the presumably less-condensed structure of 35S rDNA sites, we demonstrated that their occurrence in MN was not more frequent than 5S rDNA, suggesting that in this case factors other than DNA methylation and condensation level play a role in the inclusion of specific chromosomes in MN.

The 35S-rDNA-bearing Bd5 chromosome is characterized by having the highest levels of DNA methylation in pericentromeric regions, which decreases towards both chromosome termini. This chromosome displays two different methylation patterns depending on chromosome condensation, with less-condensed Bd5 showing a considerably lower methylation level in 35S rDNA sites [28]. In contrast to the 5S-rDNA-containing MN, we did not observe differences in the rates of MN carrying 35S rDNA sites with or without 5mC signals. Our results suggest that the chromosomes with both types of the methylation pattern contribute equally to the formation of MN.

Future studies should focus on studying the factors determining the presence of methylated or unmethylated sequences in the MN. It would be also interesting to find a connection between the DNA methylation level of MN and their future fate, i.e., whether or not they are eliminated during subsequent cell cycles.

## 4. Materials and Methods

### 4.1. Plant Material, Mutagenic Treatment, and Slide Preparation

The experiments were performed with the *Brachypodium distachyon* (2*n* = 10) reference genotype Bd21. The seed material was obtained from the collection held by the United States Department of Agriculture’s National Plant Germplasm System. *B. distachyon* seeds were presoaked in tap water for 6 h. Then the seeds were germinated on moist filter paper in Petri dishes for 72 h in the dark at 21 °C. The seedlings were treated with 4 mM maleic acid hydrazide (MH, Sigma, St Louis, MO, USA) for 3 h in the dark at 21 °C under aerated conditions. At the same time, control seedlings were incubated for 3 h in distilled water under the same aerated conditions. After the treatment, the seedlings were washed three times in distilled water and then grown in Petri dishes. The experiment with MH treatment was repeated two times. Whole seedlings were fixed in 3:1 (*v*/*v*) methanol:glacial acetic acid at three post-treatment times: 0 h, 10 h, and 20 h after treatment and stored at −20 °C until use. Cytogenetic preparations were made using a previously described procedure [56]. To make cytogenetic preparations, the root meristems were washed in 0.01 mM citrate buffer for 3 × 5 min and then digested in a mixture of maceration enzymes: 1% pectinase (*v*/*v*, Sigma) and 2% cellulose (*w*/*v*, Sigma) for 1.5 h at 37 °C. The roots were washed again in the citrate buffer and then nuclei preparations were made using 45% acetic acid. Slides were incubated on dry ice to remove the coverslips and then stored at 4 °C.

### 4.2. Immunodetection of 5-Methylcytosine

The immunodetection of 5-methylcytosine (5mC) was carried out as previously described for *B. distachyon* [12]. DNA denaturation was performed prior to the immunodetection of 5mC within nuclei and micronuclei. For the chemical denaturation of DNA, the preparations were soaked in 0.25 M sodium hydroxide (NaOH, Merck) and 1 M sodium chloride (NaCl, POCH) for 30 min at 4 °C. Then, the slides were washed 3 × 5 min in cold distilled water, and then in 1 M Tris-HCl (VWR) for 30 min at 4 °C. The preparations were washed in an alcohol series (70%, 90%, and 100%) and allowed to dry. Blocking serum (5% BSA, Sigma) in 1 × PBS was applied to the slides and incubated at room temperature (RT) in a humid chamber for 1 h. Then, the primary mouse anti-5mC antibody (Abcam, Cambridge, UK) (1:100 in 5% BSA) was applied to the slides and left in a humid chamber overnight at 4 °C. The preparations were washed 3 × 5 min in 1 × PBS. Then a secondary goat anti-mouse IgG antibody, conjugated with Alexa Fluor 488 (Invitrogen, Molecular Probes, Eugene, OR, USA), was applied to the slides and left in a humid chamber for 1 h at 37 °C. The slides were washed again for 3 × 5 min in 1 × PBS. The preparations were mounted and counterstained in Vectashield (Vector Laboratories, Peterborough, UK) that contained 2.5 µg/mL DAPI (Serva).

### 4.3. Probes Labeling and FISH Procedure

The labeling of the probes and FISH were performed according to the methodology described by Jenkins and Hasterok [56]. Two probes were used: 5S rDNA and 25S rDNA (Roche Diagnostics, Basel, Switzerland), which were labeled with digoxigenin-11-dUTP (Roche Diagnostics) and tetramethylrhodamine-5-dUTP (Roche Diagnostics) by the nick-translation method using nick-translation mix (Roche Diagnostics).

Initially, RNAse was applied to the slides and incubated at 37 °C for 1 h in a humid chamber. After this time, the slides were washed 3 × 5 min in 2 × SSC and then transferred to 1% formaldehyde (*v*/*v*) in 1 × PBS for 10 min at RT, and washed again 3 × 5 min at 2 × SSC at RT. For DNA denaturation and hybridization, a mix consisting of 50% formamide, 10% dextran sulphate (*w*/*v*), 2 × SSC, 0.5% (*w*/*v*) SDS, 75–200 ng of the DNA probes/slide, and water was applied to the slides. The slides and the mix were incubated at 70 °C for 4.5 min. The slides were cooled to 37 °C and stored overnight at 37 °C. Post-hybridization washing was performed in 20% *v*/*v* formamide in 0.1 × SSC at 42 °C for 2 × 5 min, then in 2 × SSC at 42 °C for 3 × 5 min, and finally in 2 × SSC at RT for 3 × 5 min. Then the slides were washed in Tween20/4 × SSC at RT. A blocking agent (5% milk) was applied to the slides and incubated for 30 min at RT in a humid chamber. Digoxigenated probe immunodetection was performed with a FITC-conjugated anti-digoxigenin antibody (Roche Diagnostics) and milk solution (1:11) applied to the slides and incubated for 1 h at 37 °C in a humid chamber. Then the slides were washed 3 × 8 min in Tween20/4 × SSC at 42 °C. The preparations were dehydrated in 70%, 90%, and 100% ethanol for 1 min each and allowed to dry. The air-dried preparations were mounted and counterstained in Vectashield (Vector Laboratories) containing 2.5 μg/mL DAPI (Serva, Heidelberg, Germany).

### 4.4. Image Acquisition

After 5mC immunodetection, slides were analyzed using a Carl Zeiss Imager Z2 fluorescence microscope with fluorescent lighting HSP, 120 W. The images were recorded with an AxioCam ICc5 digital camera and immersion lens with a ×100 magnification. The specific location of the cell nuclei with the micronuclei was also recorded. After subsequent FISH, the same slides were analyzed using the same equipment. The specific locations for each previously recorded cell nucleus and micronucleus were found after FISH and hybridization signals were captured. The frequencies of MN with specific signals and without signals were calculated. For the group treated with MH, a total of 350 nuclei with MN were evaluated. In order to analyze the presence of DNA methylation signals within the rDNA foci, 50 MN with rDNA signals were evaluated.

## 5. Conclusions

In this study, we presented the analyses of DNA methylation in the rDNA sites in *B. distachyon* micronuclei (MN) induced by treatment with maleic acid hydrazide (MH). This is the first cytological study of DNA methylation within specific DNA sequences in plant mutagenesis. To conclude, our results indicated that: (1) 5S and 35S rDNA in the MN can be either methylated or not; (2) the frequency of MN carrying only 5S rDNA (no 35S rDNA) without 5mC signals in this region was two times higher than the frequency of those with a 5mC signal; (3) no differences in the frequency of MN bearing methylated or unmethylated 35S rDNA were observed. Analyses of DNA methylation within other specific DNA sequences would provide more data to establish the involvement of DNA methylation in the incorporation of various sequences into micronuclei and their subsequent elimination from the genome.

## Data Availability

Not applicable.
